# Iterative Forecasting of Financial Time Series: The Greek Stock Market from 2019 to 2024

**DOI:** 10.3390/e27050497

**Published:** 2025-05-04

**Authors:** Evangelos Bakalis, Francesco Zerbetto

**Affiliations:** Dipartimento di Chimica “G. Ciamician”, Università di Bologna, V. P. Gobetti 85, 40129 Bologna, Italy; francesco.zerbetto@unibo.it

**Keywords:** *α*-Lévy stable motion, *α*-Lévy stable noise, generalised moments method, iterative stochastic differential equations

## Abstract

Predicting the evolution of financial data, if at all possible, would be very beneficial in revealing the ways in which different aspects of a global environment can impact local economies. We employ an iterative stochastic differential equation that accurately forecasts an economic time series’s next value by analysing its past. The input financial data are assumed to be consistent with an α-stable Lévy motion. The computation of the scaling exponent and the value of α, which characterises the type of the α-stable Lévy motion, are crucial for the iterative scheme. These two indices can be determined at each iteration from the form of the structure function, for the computation of which we use the method of generalised moments. Their values are used for the creation of the corresponding α-stable Lévy noise, which acts as a seed for the stochastic component. Furthermore, the drift and diffusion terms are calculated at each iteration. The proposed model is general, allowing the kind of stochastic process to vary from one iterative step to another, and its applicability is not restricted to financial data. As a case study, we consider Greece’s stock market general index over a period of five years, from September 2019 to September 2024, after the completion of bailout programmes. Greece’s economy changed from a restricted to a free market over the chosen era, and its stock market trading increments are likely to be describable by an α-stable L’evy motion. We find that α=2 and the scaling exponent *H* varies over time for every iterative step we perform. The forecasting points follow the same trend, are in good agreement with the actual data, and for most of the forecasts, the percentage error is less than 2%.

## 1. Introduction

A range of companies, securities, and other economic indices are combined to form a stock market’s general composite index (GCI). It is a marker that can convey a lot of information in an intelligible manner, and market performance can be evaluated by observing its behaviour. Working with financial data, if at all possible, it would be ideal to forecast the values of this index and thus its rate of change [1].

A financial time series is made up of the daily values of a GCI, but it is not only limited to those with a time lag of one day. Additional financial time series can be constructed by adopting a different time lag between two consecutive values. Assume that the opening or closing price of GCI on day *i* is $x_{i}$. The logarithmic difference of $x_{i}$ over two consecutive days is $r_{i}=log\left(\frac{x_{i}}{x_{i-1}}\right)$, which is the definition of the daily price return. One of the most important factors for stock market index analysis is volatility, assuming that stock market indexes are random variables [2]. Volatility measures dispersion around a central tendency and is commonly defined as the deviation from the mean. It is calculated as the variance of the daily price return, and it is a second-order statistic. As such, given that many random processes have fat tails or no mean due to outliers, its applicability is called into question [3]. However, volatility remains one of the most used methods in researching financial time series in conjunction with entropic contributions [4,5].

Financial markets can be viewed as complex, dynamic systems for which a wealth of data are available. By applying ideas from physics, financial time series can be viewed from a different perspective, potentially producing new discoveries [6]. Finding scaling exponents is a fundamental physics task in stationary or non-stationary stochastic processes. In fact, the search for these exponents in financial markets is presently a very rich field [7,8,9,10,11,12], where the growing body of empirical data is constantly enhancing our comprehension of their behaviours.

The mathematical foundation for the rescaled analysis was established by Mandelbrot and Wallis using a heuristic graphical method [13]. They demonstrated that a scaling property for stationary processes permits a relationship between past and future occurrences. Let x(t) be a stationary time series and τ be the length of the window that correlates differences between past and future events. Then, if there is a unique scaling, the formula $x\left(\tau \right)=c\tau^{H}$ holds true, where *c* is a positive constant and *H* is the scaling exponent, also known as the Hurst exponent in honour of Harold Edwin Hurst [14]. The Hurst exponent, H∈(0,1), is used to characterise a stochastic process. The process is anti-persistent for $0<H<\frac{1}{2}$, which means that each new value is likely to go in the opposite direction of the one before it. The process is referred to as persistent for $\frac{1}{2}<H<1$, and each new value probably follows the path of the preceding one. The process is memoryless for $H=\frac{1}{2}$, and each new value is completely independent of the preceding values. For financial time series, a value of H>0.5 reflects an economy at a low stage of development, as opposed to H<0.5, indicating an economy at an advanced stage of development. A financial time series is deemed monofractal if it is characterised by a single value of the Hurst exponent, but usually financial time series are better described as multifractals since they are described by multiple scalings [9,12]. Over time, numerous models have been developed in an attempt to pinpoint traits of stochastic processes, and they have been applied to the analysis of data in a range of scientific fields, including finance [3,6,15,16,17,18,19,20].

The finding of the scaling exponent of a self-similar process (Hurst exponent) is a widely used approach in econophysics [6,8] to understand the dependence of financial time series data on one another and the volatility of the stock market [13]. Back in 1963, Mandelbrot proposed an α-Lévy stable distribution as a suitable model for analysing price differences or logarithmic returns [7]. An α-Lévy stable distribution serves as a general framework for other widely used distributions, such as the log-normal that Scholes and Black [2] suggested for stock price forecasting. The latter, also referred to as Geometrical Brownian Motion (GBM), is once again α-Lévy stable with values of α=2 and H=0.5, and it is a memoryless process. A statistical analysis of the Milan, Italy, stock exchange indices revealed that the price indices are well described by α-Lévy stable distributions because the financial data’s properties are compatible with a Lévy random walk [21]. Moreover, the analysis of European options data revealed that their multifractality can be attributed to an α-stable Lévy distribution [10]. As a consequence of real data analyses, fractional Brownian motion (fBm) [22] and fractional Lévy motion (fLm) [23] have been used as row models in the analysis of economic data, showing that financial data are more appropriately described as multifractals than monofractals (fBm) [10,23,24,25,26,27,28].

Employing an α-Lévy stable distribution as a model from which the increments or the logarithmic returns draw values, it is crucial to calculate from the row data the α index and the scaling exponent $H-\frac{1}{2}+\frac{1}{\alpha }$ [26]. The Hurst exponent can be calculated using a variety of techniques, such as power spectral density (PSD) [29], rescaled range analysis (R/S) [13], detrended fluctuation analysis (DFA) [16], dispersional analysis (DA) [30], multifractal detrended fluctuation analysis (MF-DFA) [17,31], generalised Hurst exponent (GHE) [3,32], and the generalised moments method (GMM) [19,33,34], to name some of them. The accuracy of each approach in delivering the scaling exponent depends on the length of the data and the type of distribution the data satisfy (whether or not heavy tails exist). Since PSD, DFA, R/S, and DA generate a unique scaling exponent (monofractality), it is unclear if they can “capture” the features of a α-stable Lévy distribution. Furthermore, R/S has been shown to overestimate the genuine Hurst exponent [35]. Additionally, GHE performs the best, while MF-DFA is unsuitable for data with smaller sample sizes and heavier tails [18].

In this article, we outline a path that can result in financial time series forecasting using an iterative scheme. A stochastic differential equation with a drift and a diffusion term serves as the starting point [2,27,28,36]. The drift term can be calculated as the mean of the increments of the process weighted by the actual value of the process. The calculation of the diffusion term is more demanding since it requires the autocorrelation of the underlying noise originating from the discretisation of an α-Lévy stable motion. We use the GMM method [19,33,34] to classify the type of motion. It is robust in providing the scaling exponent and is similar to the generalised Hurst exponent method (GHE), which is commonly used for financial data analysis [3]. In addition, GMM is one of the most reliable techniques for analysing non-stationary time series [19], and it also performs well for short time series [18,37]. The structure function [20], whose form yields the values of the Hurst exponent and the index α, is the method’s strength, aside from the fact that it may be applied even to short data sets. For each forecast, let the trading day be the *k*th GMM analysis of the previous k−1 values. The computed α and *H* are then used to generate the α-Lévy stable noise sequence of equal length and zero mean, whose value, after numerous iterations, contributes to the actual value of the index for the day *k*. The values of α and *H* may vary at every prediction point. It is important to note that as part of the data preprocessing procedure, we calculate the historical data sample entropy [38,39], whose value can reveal whether the time series behaves as a scale-free process [40] or is predictable or regular.

The current work is organised as follows: The application of the generalised moments approach to time series and the retrieved data is covered in Section 2.1. Section 2.2 discusses fractional Lévy stable motion and self-similar processes. Stochastic differential equations are covered in Section 2.3. Section 3 presents the results and discussions, and Section 4 summarises.

## 2. Materials and Methods

### 2.1. Time Series and Generalised Moments Method

Let $\left\{x_{i}\right\}$ with i=1,2,…,N be a process or the measure of a field we study. We are interested in understanding how the fluctuations of the field behave, and for this purpose we define the increments of the field. In order to reveal scaling properties, where scaling is defined as the invariance of a process’s properties across a range of scales, we create many intermediate scales, for example, by dividing the field into non-overlapping intervals of certain resolution or time lag Δ, and producing new processes defined as $y_{n}\left(\Delta \right)=\left|x\left(n+\Delta \right)-x\left(n\right)\right|$. Scaling can be described using both fractal and multifractal theories. A unique scaling, or monofractal, is the focus of fractal theory [41] and only a few parameters are required to describe complex phenomena. Nonetheless, multifractal theory addresses multiscaling, which enables the scaling characteristics of a process to be generalised [42,43]. The scaling properties of the field can be determined either by the scaling of its probability distribution, $Pr\left(y_{\Delta }>\Delta^{\alpha }\right)∼\Delta^{-f(\alpha )}$ or equivalently by the scaling of its different moments scaling function $<y_{\Delta }{>}^{q}∼\Delta^{z(q)}$ [20].

The scaling exponent z(q), also referred to as the structure function, determines whether a process is monofractal (has a linear dependence of z(q) on *q*) or multifractal (has a convex shape). Among multifractals, universal multifractals, which essentially are a log-Lévy multifractals, are ubiquitous [20], and the scaling exponent reads

(1) $$z\left(q\right)=qh-\frac{C}{\alpha -1}\left(q^{\alpha }-q\right)$$

where $\alpha =-\frac{1}{C}\frac{d^{2}z\left(q\right)}{dq^{2}}{|}_{q=1}$ is the Lévy index or index of multifractality. It takes values in the interval 0<α≤2. For α=2, $z\left(q\right)=qh-C\left(q^{2}-q\right)$, the logarithm of the field is normally distributed [44], and Equation (5) depicts the characteristic function of a Gaussian distribution. For α=1, z(q)=qh−Cqlog(q), and the field is distributed according to log-Cauchy. For all the other values of α in the range (0, 2), the field is distributed according to log-Lévy. The term *C* is called co-dimension information and measures the mean intermittency and is defined as $C=\frac{dz(q)}{dq}{|}_{q=1}-H$. It takes values in the range C∈[0,d], with *d* being the dimension of the support, which is 1 for one-dimensional time series. For C=0, the field is homogeneous; only one scale exists. From lower *C* to higher values of *C*, the degree of intermittency increases, and some extreme outliers will occur. The parameter h∈(0,1), when the field is multifractal, shows the degree of fractional integration (persistent for h>0.5 and antipersistent for h<0.5). The value of z(q) for q=1 provides the Hurst exponent for a multifractal field. Since z(q)=qh, it follows that z(q=1)=z(q=2)/2 when a field is monofractal [45]. Therefore, the analytical form of the scaling exponent or structure function, Equation (1), gives information about the nature of the stochastic mechanisms; see, for details, refs. [19,31,33,34,45,46,47].

Let us assume that $\left\{x_{n}\right\}$ is a financial time series with the index n=1,2,…,N to stand for the day, and *N* is the total number of recordings. We consider here as a minimum time lag one day, but it can also be smaller or greater, depending on the type of recordings. If $\left\{x_{n}\right\}$ is a self-similar process, then it is fully described by the scaling exponent (Hurst) [22,23]. We expect that such processes, when zoomed in or zoomed out, will reveal the same patterns scaled by a certain amount, $\left\{x_{an}\right\}\overset{d}{=}\left\{a^{H}x_{n}\right\}$, where $\left\{..\right\}\overset{d}{=}\left\{..\right\}$ stands for the equality of finite dimensional distributions, and *H* is the scaling exponent. Let *t* and *s* with (t>s) be two distinct time moments, then it can be pointed out that $∥x\left(t\right)-x\left(s\right)∥∼{\left|t-s\right|}^{H}$; see ref. [31] for the proof. Taking into account that the various moments can scale differently, we write ${∥x\left(t\right)-x\left(s\right)∥}^{q}∼{\left|t-s\right|}^{qH(q)}$, with H(q) being the generalised Hurst exponent through which the structure function is defined z(q)=qH(q). For discrete data sets, the time difference t−s describes the time lag whose maximum value should be small with respect to the total length of the time series; one tenth of the total length is a safe choice. For the application of the GMM, we follow the steps:

(i) The construction of time series characterized by different lag times, Δ, which contain the absolute change of the values between two points of the initial time series $y_{n}\left(\Delta \right)=∥x\left(n+\Delta \right)-x\left(n\right)∥$ for n=1,2,…,(N−Δ) and for $\Delta =1,2,\ldots ,\frac{N}{10}$, where $\Delta =\frac{N}{10}$ is the maximum lag time.
(ii) Calculation of the moments of various orders *q* for q>0, including also fractional orders $m\left(q,\Delta \right)=\frac{1}{T-\Delta }\sum_{n=1}^{T-\Delta }y_{n}{\left(\Delta \right)}^{q}$. Notice that the small order moments, 0<q<2, are responsible for the core of the probability density function (pdf), and higher moments contribute to tails of a pdf [48].
(iii) Finding the scaling exponent for each moment. Assuming that $m\left(q,\Delta \right)∼\Delta^{qH(q)}$, then the slope of the linear regression in log–log scale returns the value of H(q) for each *q*.
(iv) Construction of the structure function. Having the values of the term qH(q) for the various moments, fit the data with Equation (1) and find the parameters H,α,C.

### 2.2. Self-Similar Stochastic Models and Fractional Lévy Stable Motion

Typically, random processes are not stationary, as opposed to those that are stationary, for which it holds true that $\left(x\left(t+b\right)-x\left(t\right)\right)\overset{d}{=}\left(x\left(b\right)-x\left(0\right)\right)$∀b≥0 with ${\left\{x\left(t\right)\right\}}_{t\geq 0}$. Those with stationary increments are a significant class among them. Fractional Brownian motion $B_{H}\left(t\right)$ (fBm) is the term used to describe a non-stationary and self-similar process whose increments are stationary and also derived from a Gaussian distribution as defined by Mandelbrot and van Ness [22]:

(2) $$B_{H}\left(t\right):=\frac{1}{\Gamma (H+\frac{1}{2})}\left(\int_{0}^{t}{\left(t-\tau \right)}_{+}^{H-\frac{1}{2}}dB\left(\tau \right)+\int_{-\infty }^{0}\left({\left(t-\tau \right)}_{+}^{H-\frac{1}{2}}-{\left(-\tau \right)}_{+}^{H-\frac{1}{2}}\right)dB\left(\tau \right)\right)$$

where ${\left(x\right)}_{+}=max\left(x,0\right)$. Since input data are typically discrete rather than continuous, it is preferable to create a process that contains the increments of the initial process. The one-step increment for a continuous fBm is called fractional Gaussian noise $b_{H}\left(t\right)$ (fGn). It is a self- similar stationary process; its sum up to the *n*th event yields the fBm valid at that time, and it is defined as

(3) $$b_{H}\left(t\right)=\frac{1}{\Gamma (H+\frac{1}{2})}\int_{-\infty }^{t}\left({\left(t+1-\tau \right)}^{H-\frac{1}{2}}-{\left(t-\tau \right)}^{H-\frac{1}{2}}\right)w\left(\tau \right)d\tau$$

with w(t) being a collection of independent and identically distributed (i.i.d.) Gaussian random variables (white noise), and Γ() being the Euler’s gamma function. The autocovariance function of fGn reads

(4) $$<b_{H}\left(t\right)b_{H}\left(t^{\prime }\right)>=\frac{\Gamma (1-2H)cos(\pi H)}{2\pi H}\left\{{\left(t-t^{\prime }+1\right)}^{2H}+{\left(t-t^{\prime }-1\right)}^{2H}-2{\left(t-t^{\prime }\right)}^{2H}\right\}$$

The properties of fGn depend on the definition of fBm. There are three definitions, Kolmogorov, Lévy, and Mandelbrot van Ness (see Equation (2) for the latter), and they do not share the same properties [49,50]. If a process is characterised as fBm, then the structure function, Equation (1), takes the simple form z(q)=qH, which easily determines the Hurst exponent [19,33,45]. In addition, the increments of fBm are drawn from a Gaussian distribution which serves as a mother process to generate the various fGn or fBm under integration/differentiation [51]. Gaussian distributions are stable distributions that are maintained under linear transformations and have been demonstrated to be effective models for highly variable data [23].

The term “stable” refers to a significant class of probability distributions with intriguing characteristics, in which any linear combination of two i.i.d. random variables with a common distribution can be linearly transformed to have the same distribution [23,24]. Stable distributions are also known as α-stable Lévy distributions. An α-stable distribution, $L_{\alpha }\left(X;\mu ,\beta ,\sigma \right)$, requires four parameters for its complete description. α∈(0,2] is the index of stability or Lévy index. There is a greater chance of outliers and heavier tails when α takes low values. β∈[−1,1] is a skewness parameter; a left-skewed distribution has a negative value of β, while a right- skewed distribution has a positive value. For β=0, the distribution is symmetric, and its characteristic function describes a stretched exponential centred around its mean or location parameter μ∈R; see Equation (5) below. Finally, $\sigma \in R_{+}^{*}$ is a scale parameter. The probability density function is merely scaled and offset by the scale and location parameters; refer to ref. [24] for additional information on stable distributions. For α<2, the distribution has undefined variance, while for α≤1, it has an undefined mean. For α=2 and for α=1, the normal and the Cauchy distributions are returned.

An α-stable distribution is defined through its characteristic function Φ(k), which is the Fourier transform of the probability distribution, $\Phi \left(k\right)=P\left(k;\alpha ,\beta ,\mu ,\sigma \right)=\int_{-\infty }^{\infty }e^{ikX}P\left(X;\alpha ,\beta ,\mu ,\sigma \right)$ and always exists for any real-valued random variable as opposed to the moments generating function. The Φ(k) of an α-stable distribution reads [23]

(5) $$ln\left(\Phi \left(k\right)\right)=\left\{\begin{array}{cc} -\sigma^{\alpha }{\left|k\right|}^{\alpha }\{1-i\beta sign\left(k\right)tan\left(\frac{\pi \alpha }{2}\right\}+i\mu k & \alpha \neq 1\\ -\sigma |k|\{1+i\beta sign\left(k\right)\frac{2}{\pi }ln|k|\}+i\mu k & \alpha =1 \end{array}\right.$$

The symmetric α-stable Lévy motion, also known as Lévy stable motion (Lsm) $L_{\alpha }=\{L_{\alpha }\left(t\right),t\geq 0$, is a Markovian stochastic process that starts at 0. It has stationary increments, and it is self-similar $L_{\alpha }\left(kt\right)\overset{\Delta }{=}k^{1/\alpha }L_{\alpha }\left(t\right)$ with scaling exponent H=1/α. For α=2, Lsm returns the ordinary Brownian motion $L_{2}\left(t\right)\overset{\Delta }{=}B\left(t\right)$ (actually $L_{2}\left(t\right)=\sqrt{2}B\left(t\right)$). Similar to the generalisation of Brownian motion to fractional Brownian motion (fBm) by Mandelbrot and van Ness [41], we define the fractional Lévy stable motion (fLsm) as the following integral [23]:

(6) $$L_{\alpha }^{H}\left(t\right)=\int_{-\infty }^{\infty }\left\{cf_{\alpha ,+}^{H}\left(t,\tau \right)+df_{\alpha ,-}^{H}\left(t,\tau \right)\right\}M\left(d\tau \right)$$

where $f_{\alpha ,+}^{H}\left(t,\tau \right)={\left(t-\tau \right)}_{+}^{H-1/2}-{\left(-\tau \right)}_{+}^{H-1/2}$,$f_{\alpha ,-}^{H}\left(t,\tau \right)={\left(t-\tau \right)}_{-}^{H-1/2}-{\left(-\tau \right)}_{-}^{H-1/2}$, ${\left(x\right)}_{+}=max\left(x,0\right)$, ${\left(x\right)}_{-}=min\left(x,0\right)$, *M* is an α-stable random measure in *R*. fLsm is a self-similar with stationary increments process (H-sssi) $L_{\alpha }^{H}\left(kt\right)=k^{\gamma }L_{\alpha }^{H}\left(t\right)$ with $\gamma =H-\frac{1}{\alpha }$ and $H\in [\frac{1}{\alpha },1)$ [36,52]. For H=1/α, fLsm becomes an ordinary α-stable Lévy motion (Lsm) $L_{\alpha }\left(t\right)$. The self-similarity exponent of Lsm lies in the range $\left(\frac{1}{2},\infty \right)$, and for 1≤α<2, Lsm is not the only self-similar process with stationary increments, as opposed to 0<α<1 and α=2, where it is the only one [23]. The increments of fLsm, Equation (6), form the fractional Lévy noise $l_{\alpha }^{H}\left(t\right)$ that presents long-range dependence for γ>0, and negative dependence for γ<0. For γ=0, the increments are i.i.d. symmetric α-stable variables:

(7) $$l_{\alpha }^{H}\left(t\right)=L_{\alpha }^{H}\left(t+1\right)-L_{\alpha }^{H}\left(t\right)$$

A process driven by fLsn is anticipated to not have moments for q>α. However, for finite-size discrete data sets, all moments, independent of the order *q*, exist. As a result of that, Equation (1) can be used to deliver the detailed form of the structure function. It has been argued that when z(q=1) is different from z(q=2)/2, any additive model cannot be applied, and unavoidably, multifractal behaviour emerges [20]. However, such a result can be understood as being a bi-fractal behaviour result of a truncated Lévy motion, either standard or fractional [53].

### 2.3. Stochastic Differential Equation

The stochastic differential equation for Lévy stable motion is given by [23,54]:

(8) $$dx_{\alpha }\left(t\right)=\mu \left(t,x_{\alpha }\left(t\right)\right)dt+\sigma \left(t,x_{\alpha }\left(t\right)\right)dL_{\alpha }\left(t\right)$$

where $x_{\alpha }\left(t=0\right)=x_{\alpha ,0}$ is a random variable that takes values from an α-Lsm, the measure μ(t,x(t)) describes the drift term, and σ(t,x(t)) stands for the diffusion term. Equation (8) can also be presented in integral form

(9) $$x_{\alpha }\left(t\right)=x_{\alpha ,0}+\int_{0}^{t}\mu \left(\tau ,x_{\alpha }\left(\tau -\right)\right)d\tau +\int_{0}^{t}\sigma \left(\tau ,x_{\alpha }\left(\tau -\right)\right)dL_{\alpha }\left(\tau \right),t\geq 0$$

In line with the derivation of the stochastic differential equation for fractional Brownian motion [55], and its integral representation, Equation (9), which is based on fractional Ito’s formula [27], the integral form of a process driven by an α-fLsm is written as [28]

(10) $$x_{\alpha }^{H}\left(t\right)=x_{\alpha ,0}^{H}+\int_{0}^{t}\mu \left(\tau ,x_{\alpha }^{H}\left(\tau -\right)\right)d\tau +\int_{0}^{t}\sigma \left(\tau ,x_{\alpha }^{H}\left(\tau -\right)\right)dL_{\alpha }^{H}\left(\tau \right),t\geq 0$$

where $x_{\alpha ,0}^{H}$ is a random variable drawing values from an α-fLsm. The differential form of Equation (10) is given by an equation like Equation (8), where we replace $x_{\alpha }\left(t\right)$ with $x_{\alpha }^{H}\left(t\right)$ and $dL_{\alpha }\left(t\right)$ with $dL_{\alpha }^{H}\left(t\right)$. It should be noted that the integral of the stochastic term in both Equations (9) and (10) requires careful handling [28,56].

The drift and diffusion terms, in a first approximation, can be expressed as μ(t,x(t))=μx(t) and σ(t,x(t))=σx(t), respectively. Consequently, we can express in discrete space a stochastic differential equation that can prospectively forecast future values using an iterative scheme, but we require an estimate of μ and σ to do so. Let ξ be a generic process that takes values from a probability distribution; it can represent fLsm, fbm, or multifractional Brownian motion (mfBm). For ξ, the discrete equivalent of Equation (8) is as follows:

(11) Δx(t)=μx(t)Δt+σx(t)Δξ(t)

where Δt is the minimum time lag related to the resolution of the available data set. Moreover, we consider that ξ(t) has stationary increments with zero mean, <Δξ(t)>=0, and variance that remains to be specified and is related to the type of the process. Notice that we can also write $\Delta \xi \left(t\right)=w_{\xi }\left(t\right){\left(\Delta t\right)}^{s}$, where $w_{\xi }\left(t\right)$ is a white noise that draws values from ξ(t) and *s* is the self-similarity exponent, *H* for fBm, $H-\frac{1}{2}+\frac{1}{\alpha }$ for fLsm, and H(t) for mfBm [27]. We divide each term of Equation (11) by x(t) and write

(12) $$\frac{\Delta x(t)}{x(t)}=\mu \Delta t+\sigma \Delta \xi \left(t\right)$$

Taking the expectation value of Equation (12), and since <Δξ(t)>=0, we find

(13) $$\mu =<\frac{\Delta x(t)}{x(t)}>=\frac{1}{N}\sum_{n=1}^{N}\frac{\Delta x(t_{n})}{x(t_{n})}$$

with *N* being the length of the data at time t. In addition, we calculate the expectation of the term $<\frac{\Delta x(t)}{x(t)}\frac{\Delta x(t^{\prime })}{x(t^{\prime })}>$ and we find that that equals to $\mu^{2}+\sigma^{2}ACF_{\Delta \xi }(|t-t^{\prime }\left|\right)$, where $ACF_{\Delta \xi }(|t-t^{\prime }\left|\right)$ is the autocorrelation of Δξ(t). Combining our findings, we end up with

(14) $$\sigma ={\left(ACF_{\Delta \xi }\left(0\right)\right)}^{-1/2}\sqrt{<{\left(\frac{\Delta x(t)}{x(t)}\right)}^{2}>-<\left(\frac{\Delta x(t)}{x(t)}\right){>}^{2}}$$

with $ACF_{\Delta \xi }\left(0\right)$ being the value of the autocorrelation function for $t=t^{\prime }$. Equation (11) for a generic stochastic motion ξ(t) in conjunction with Equations (13) and (14) is the main equation utilised in the iterative forecast.

## 3. Results and Discussion

As a case study, we consider Greece’s stock market general index over a period of five years, from September 2019 to September 2024, after the completion of bailout programmes. Greece’s economy changed from a restricted to a free market over the chosen era, and its stock market trading increments are likely to be describable by an α-stable L’evy motion. For the period between 4 September 2019, and 6 September 2024, we examine the Athens General Composite (ATG) index. The website https://tradingeconomics.com/greece/stock-market (accessed on 10 September 2024) is where the data were obtained.

**Figure 1:**
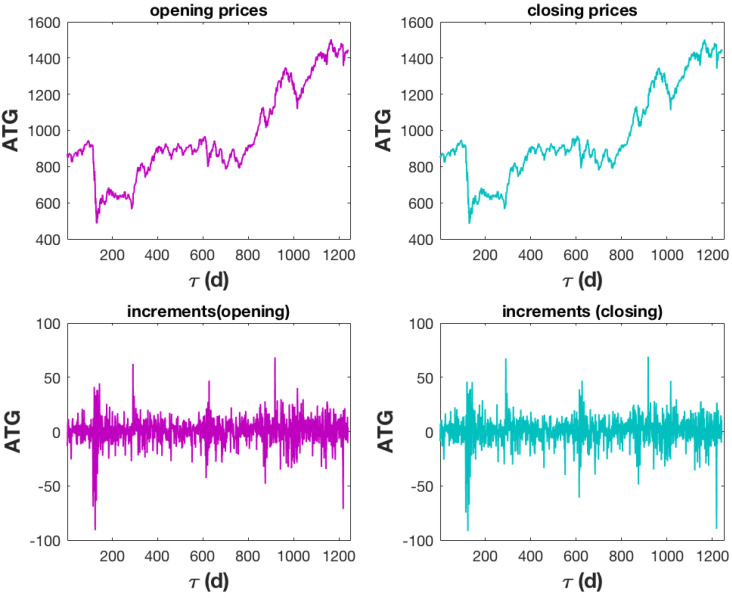
The upper panel displays the ATG’s (Athens General Index) opening and closing prices over a five-year period, from September 2019 to September 2024. Although these days are not always calendar consecutive, two consecutive events indicate two trading days. The bottom panel shows the increments of the processes shown in the upper panel. The website https://tradingeconomics.com/greece/stock-market (accessed on 10 September 2024) is where the data were obtained.

The trend of ATG as well as its increments are displayed in Figure 1: the upper left panel for the opening prices, the upper right panel for the closing prices, the bottom left panel for the increments of the opening prices, and the bottom right panel for the increments of the closing prices. Opening and closing prices are extremely similar and correspond to non-stationary processes.

The characteristics of a non-stationary time series alter over time. Trends, cycles, and random events derived from one or more probability distributions in an additive or multiplicative mode can all be found in a non-stationary time series. The degree of randomness that defines a time series is what remains after trends are eliminated by subtracting either the mean or the moving average mean, and cycles, if any, can be handled similarly.

**Figure 2:**
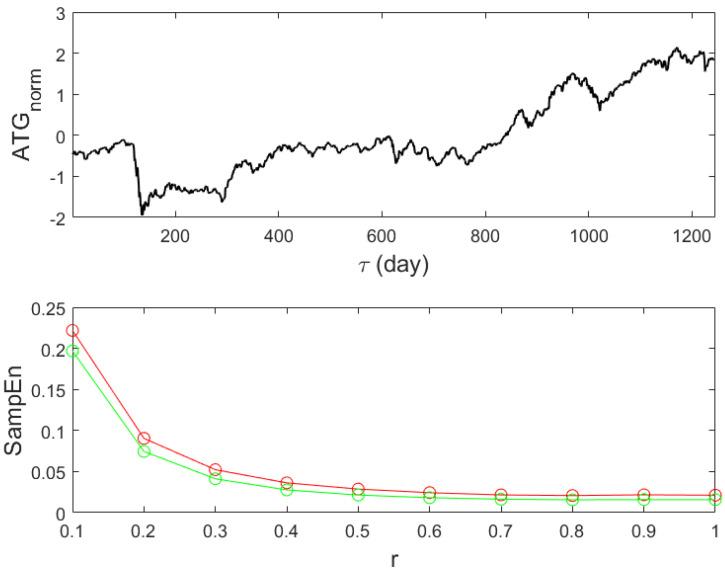
The normalised opening prices of ATG, see also Figure 1 for comparison, are displayed in the upper panel. Its values serve as input for the sample entropy calculation (bottom panel). Two distinct values of *m* (red for *m* = 1 and green for *m* = 2) and a range of tolerance *r* values are used to estimate the sample entropy.

The sample entropy (SampEn) value for a time series indicates how predictable or regular the time series is; low values indicate a more predictable system, while high values indicate randomness or chaos. Originally developed for physiological applications [38], it finds applications in a variety of fields, including finance [39]. The algorithm of SampEn draws inspiration from the embedding dimensions formulated by Grassberger and Procaccia in the early eighties [57,58]. Details of the algorithm and a comparison with the similar but not same concept of approximate entropy can be found in a recent tutorial [59]. SampEn(data,m,r) [60] is the function that runs in Matlab and takes three arguments. The first one (data) stands for the normalised input, where for normalisation we consider the relation $x_{n}=\frac{x-<x>}{\sigma_{x}}$; see upper panel of Figure 2. The second argument *m* stands for the embedding dimension, and it is always smaller than the total length of the inp ut data; usually the first values (m=1,2) are of interest. The third argument *r* is the tolerance and is expressed as a fraction of the standard deviation.

In Figure 2, the bottom panel shows the value of SampEn for two different values of *m* (m=1 in red and m=2 in green) for values of tolerance between 0.1 and 1. All values of SampEn are small, indicating a predictable system, and in addition, its value becomes smaller, increasing the value of the tolerance. Especially for values of *r* greater than 0.5, the conditional probability of the two patterns to be compared is close to 1, underlying the existence of a genuine order. However, such an order, the existence of patterns, is not detected by any other method; see below. Given that tolerance is a portion of the standard deviation and that the latter is affected by outliers, the criterion of matching can be true for all compared patterns only because the dynamic scale has been altered by the outliers. Consequently, SampEn suggests that the ATG index from Sep 2019 to 2024 behaves as a scale-free system, and its scaling exponent remains to be estimated [40,61].

**Figure 3:**
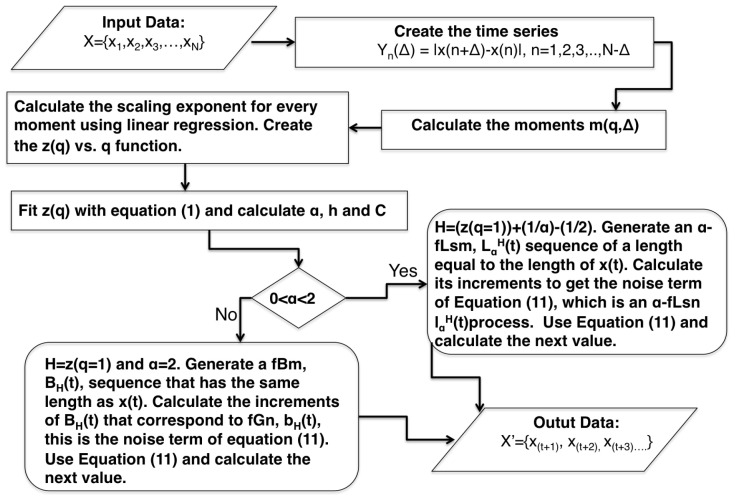
The flowchart shows the steps that need to be taken from data entry to output, which is the forecast for each subsequent trading day.

In the following, we focus only on the opening prices; the flowchart shown in Figure 3 serves as the basis for their investigation.

The input data are used iteratively to characterise a process of length *k*, with a minimum value of 0.8N The value of 0.8N is used in this work, but it can be even smaller. GMM requires trajectories of a minimum of 100 data points. and a maximum of N−1, where *N* is the total length of the trajectory depicted in Figure 1. For each given value of *k*, we apply GMM and calculate the values of α, *h*, and *C*, thus succeeding in characterising the type of process and its self-similarity exponent.

**Figure 4:**
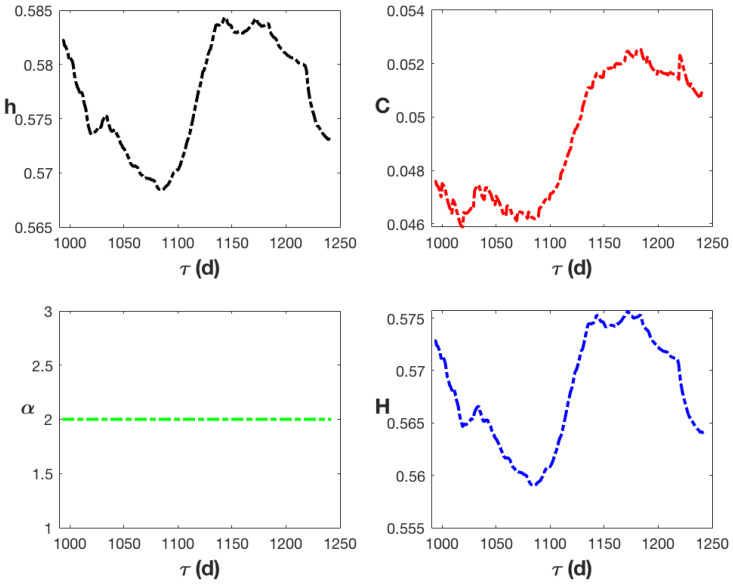
The calculated values of *h*, *C*, α, and *H* for the recorded stock market prices that cover a time window of at least 990 days to a maximum of 1240 days. Each new trading day increases the length of the recorded data by one.

The degree of intermittency (*C*) falls between 0.046 and 0.053, and the degree of fractional integration (*h*) is consistently higher than 0.5, suggesting a persistent process. Furthermore, the process draws values from a Gaussian distribution since the index of multifractality α is always 2. In the interval (0.55, 0.58), the scaling or Hurst exponent (*H*), which is the value of the structure function for q=1, fluctuates slightly from trading day to trading day. The findings depicted in Figure 4 rather suggest a process with a time-varying Hurst exponent, or mfBm [27].

To forecast the next value of the opening prices data shown in Figure 1, using Equation (11), and since the input data correspond to an mfBm as the values of α and *H* indicate and are shown in Figure 4, it is necessary to generate an fBm process every time with scaling exponent H(t), whose increments correspond to an fGn process. It is evident that in each iteration, the length of the generated fBm process increases, and its scaling exponent changes. Sequences of fBm are created using Matlab’s built-in function wfBm(H,k) with zero mean, variance 1, Hurst exponent *H*, and length *k* [62]. Next, we use the increments of fBm to generate fractional Gaussian noise sequences. For every iteration, 200 of these sequences are produced, and they yield the noise value in Equation (11).

**Figure 5:**
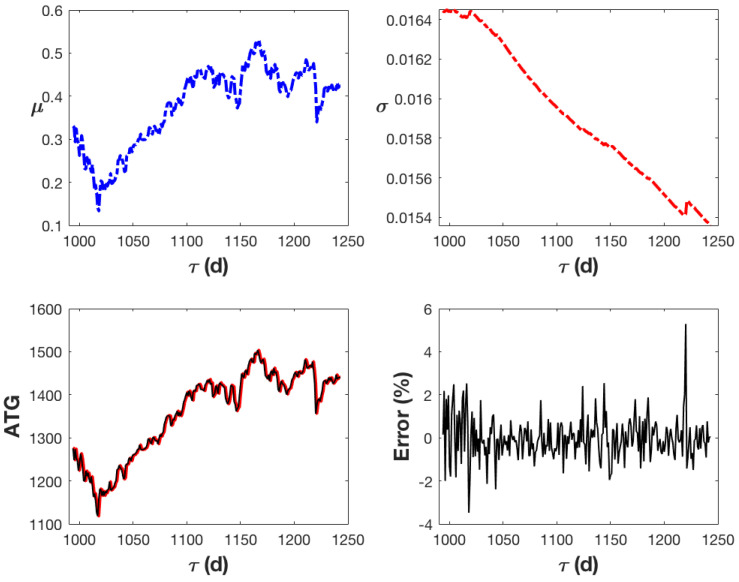
The upper panel shows how the diffusion term, Equation (14), and the mean, Equation (13), change over the course of the analysis. The ATG index’s evolution over the course of five years is shown in the bottom panel in black, and iterative predictions made using Equation (11) are shown in red. Additionally, the relative percentage error is illustrated.

**Figure 6:**
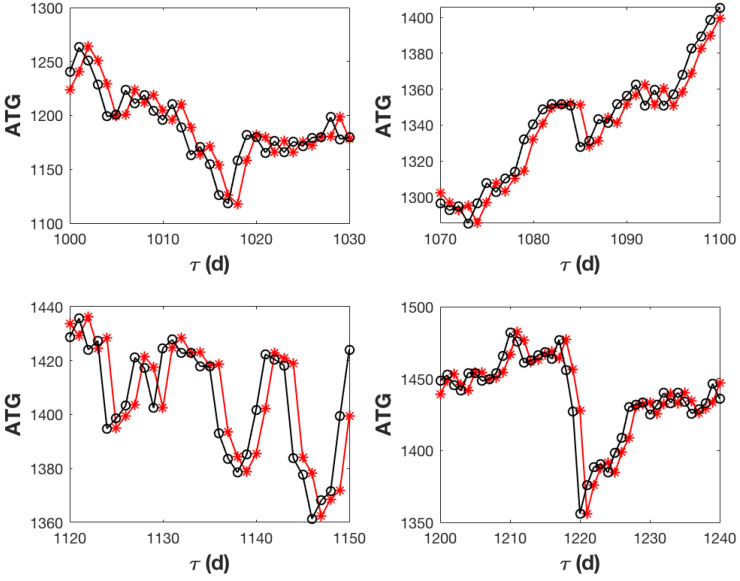
The ATG evolution is depicted in black in these snapshots of the analysed trajectory, along with the prediction, in red, made using the iterative scheme suggested in this work.

As anticipated, given that μ is weighted by the value of ATG by definition, the drift term μ of Equation (11) varies over the course of each trading day and has a form that is very similar to the dependence of ATG on time. Additionally, the diffusion term σ provided by Equation (14) exhibits a decreasing behaviour. Figure 5 displays the ATG value for each trading day (in black) along with its predictive value (in red), which is calculated using Equation (11). With a larger percentage error value of less than 6%, both curves show excellent agreement; for a higher resolution, see Figure 6.

Figure 6, in which short time windows are used to increase resolution, shows some snapshots of the ATG evolution along with the corresponding predictions. It is noteworthy that the trend of the forecasts closely resembles the actual trend of changes, and the forecast values are close to the actual values. The bulk of the forecasts have a percentage error of less than 2%, while their largest value of 6% corresponds to trading day 1220, where the recorded stock market change exceeds nearly 80 basis points. Even in this instance, the forecast demonstrates its predictive power by providing the right trend and a change of about 50 basis points.

## 4. Conclusions

In this work, we propose a model that can predict the next value of a stochastic process through iteration. There are both deterministic and stochastic terms in the fundamental equation that form the basis of the iterative action. The method of generalised moments, which enables the classification of the type of stochastic process—which we consider a priori to be an α-stable Lévy process—is essential to the model’s application. We calculate the drift term, which is an average of the process increments weighted by the process values, and the diffusion term, which includes the effect of the autocorrelation that corresponds to the original process noise, after determining the values of α and the scaling exponent *H*. We use the Athens General Index as an actual data field from September 2019 to September 2024 in order to evaluate the model. Predicted values are in line with actual data trends, predictions are satisfactory, and the extreme value of the percentage error does not exceed 6%.

## Data Availability

As mentioned explicitly in the text, the information analysed in this study was obtained from the website https://tradingeconomics.com/greece/stock-market (accessed on 10 September 2024). No additional data have been produced.

## Abbreviations

The following abbreviations are used in this manuscript:

fBm	fractional Brownian motion
fGn	fractional Gaussian noise
mfBm	multifractional fractional Brownian motion
fLsm	fractional Lévy stable motion
fLsn	fractional Lévy stable noise
